# Antibacterial activity of biodentine against *Enterococcus faecalis*: a systematic review

**DOI:** 10.3389/fdmed.2024.1498353

**Published:** 2025-01-21

**Authors:** Hasan Subhi, Nashwah Subhi, Salah Alhaidary, Mahmood S. Azeez, Abedelmalek Kalefh Tabnjh

**Affiliations:** ^1^ School of Dental Sciences, Universiti Sains Malaysia, Kelantan, Malaysia; ^2^ Department of Prosthetic Dentistry, College of Dentistry, University of Mosul, Mosul, Iraq; ^3^ Department of Orthodontics, Faculty of Dentistry, Ibn Al-Nafis University, Sana’a, Yemen; ^4^ Pharmacure Pharmacy, Azhar Private Hospital, Muscat, Sultanate of Oman; ^5^ Department of Cariology, Odontology School, Sahlgrenska Academy, Gothenburg University, Göteborg, Sweden; ^6^ Department of Applied Dental Sciences, Faculty of Applied Medical Sciences, Jordan University of Science and Technology, Irbid, Jordan; ^7^ Dental Research Unit, Center for Global Health Research, Saveetha Medical College and Hospital, Saveetha Institute of Medical and Technical Sciences, Saveetha University, Chennai, India

**Keywords:** antibacterial, biodentine, *Enterococcus faecalis*, endodontic treatment, endodontic failure, systematic review

## Abstract

**Introduction:**

Biodentine is a well-known endodontic material that is applied in various endodontic therapies. *Enterococcus faecalis* (*E. faecalis*) is associated with endodontic failure and persistent periapical infection. The purpose of this systematic review was to summarize the available evidence regarding the antibacterial activity of Biodentine against *E. faecalis* and to compare it to other commercial endodontic materials.

**Methods:**

An electronic search of literature was conducted in PubMed, Scopus, Web of Science, and Google Scholar in addition to a manual search in specialized journals up to May 2024. The eligibility criteria, data extraction, and evaluation of risk of bias were assessed by two independent authors. The risk of bias was evaluated in accordance with Modified CONSORT checklist items for pre-clinical in vitro studies on dental materials.

**Results:**

Out of 343 studies, thirty-seven fulfilled the inclusion criteria and were included in this review. Thirty studies reported a good antibacterial efficacy of Biodentine against *E. faecalis*. Biodentine was superior to or, at least, as efficacious as MTA, MTA Angelus, GIC, RMGIC, DiaRoot BioAggregate, NeoPutty, iRoot FS, MTA Repair HP, MTA Biorep, Well-Root PT, Activa, NeoMTA 2, Calcimol LC, TotalFill, and IRM. The findings were supported by studies with medium to high risk of bias (low quality).

**Conclusions:**

Considering the limitations of this systematic review, there is accumulating evidence on the antibacterial activity of Biodentine against *E. faecalis* in context of endodontics. However, randomized clinical trials with well-designed and robust methodologies are required in order to provide information about its clinical behaviour.

## 1 Introduction

The success of endodontic treatment relies on an accurate diagnosis and a definitive treatment plan (1). Oral bacteria have a significant role in the development and progression of pulpal and periapical diseases, as well as in the failure of endodontic treatment (2). Most inflammatory pulpal and periapical diseases are initially treated with conservative nonsurgical treatments (3). Inadequate cleaning of the root canal and persistent/secondary intraradicular infection attributes to re-infection of the root canal, and leads to endodontic failure (4). Surgical intervention (such as apicoectomy and retrograde filling) becomes necessary to save the tooth when nonsurgical treatments have failed (3).

Certain bacteria are frequently found in infected root-filled teeth (5). *E. faecalis* is commonly detected in the cases of failed endodontic treatment (5), with a percentage rate of 77% (6). *E. faecalis* is a facultatively anaerobic Gram-positive coccus (7) that exhibits high resistance to antimicrobial agents and tolerates low-nutrient and highly alkaline environments (7–9). In addition, it has the ability to penetrate dentinal tubules (10) and form biofilms on the root canal walls (11).

Following endodontic treatment of chronic periodontal disease, *E. faecalis* is often associated with persistent intra-radicular and extra-radicular infections (12), although recent evidence indicates that it is not considered the key pathogen in root canal infection (13). The short-term application of intracanal dressing is often insufficient in eradicating bacteria, as the medicament fails to reach the intended sites (14). Furthermore, the inflammation can be efficiently reduced by antibiotics in acute or chronic apical periodontitis (15); however, a complete relief is often hindered by the presence of unreachable bacteria within the canal system (16).

Surgical intervention is the treatment of choice if conventional endodontic treatment/re-treatment fails to save the tooth. This approach involves the removal of persistent pathogens by debridement of infected periradicular tissue, resection of root-end (apicoectomy), and obturation of retrograde root canal (root-end filling) (17). If there are remaining intracanal bacteria, the tight root-end filling will seal the apical termination of root canal and encases the remaining bacteria (18). Therefore, it is important for the root-end filling materials to have antibacterial properties.

Biodentine is one of the well-known root-end filling materials which has drawn attention in recent years. It was introduced by Septodont in 2009 as a dentin replacement material. The powder consists of tricalcium, dicalcium silicate, calcium carbonate and oxide filler, iron oxide shade, and zirconium oxide (as radiopacifier). The liquid mainly contains calcium chloride in an aqueous solution (as an accelerator) with an admixture of hydrosoluble polymer (as a water reducing agent) (19). The hydration of the calcium silicate components leads to the formation of calcium silicate hydrate and calcium hydroxide, the latter of which directly promotes antimicrobial effects (20). The studies have shown that Biodentine is biocompatible (21), stimulates odontoblast differentiation (22) and reparative dentin formation (23, 24), and has an adequate sealing ability (25). Therefore, Biodentine is considered a suitable material for application in various endodontic therapies including surgical endodontics (19).

Many studies have investigated the antibacterial activity of this material against *E. faecalis*. However, there is a conflicting overview of the existing findings to determine the effectiveness of Biodentine against *E. faecalis*. This review aims to systematically summarize the available evidence on the antibacterial activity of Biodentine material against *E. faecalis* and to compare it to other commercial endodontic materials.

## 2 Methodology

### 2.1 Review question

The following PICOS guided the formulation of the research question: P (population): Bacterial cultures of *E. faecalis*; I (intervention): Biodentine; C (comparators) commercial endodontic materials; O (outcomes) Inhibition or reduction in bacterial growth; S (study design) all relevant *in vitro* and *in vivo* studies. Based on the PICOS components, the review question is: Is Biodentine effective in inhibiting the growth of *E. faecalis*, and how does its effectiveness compare to that of other commercial endodontic materials?

The null hypothesis for this review is: Biodentine exhibits antibacterial activity against *E. faecalis* that is not significantly different from, or superior to, other commercial endodontic materials.

### 2.2 Literature search

This systematic review was conducted in accordance with the Preferred Reporting Items for Systematic Reviews and Meta-analyses (PRISMA) (26).

A comprehensive literature search was conducted in March 2024 (and updated in May 2024) in four electronic databases, PubMed, Scopus, Web of Science, and Google Scholar, for the studies published since 2009 (the date of Biodentine introduction) up to May 2024. A combination of search keywords and Medical Subject Headings (MeSH) terms with Boolean operators (AND, OR) was used, as shown in Table 1. In addition, a manual search was conducted in the following dental materials- and endodontics-related journals: International Endodontic Journal, Journal of Endodontics, Australian Endodontic Journal, Endodontology, Dental Materials, Dental Materials Journal, and Oral Surgery, Oral Medicine, Oral Pathology, and Oral Radiology, to find out articles that did not appear in the electronic search of the above database outcome.

**Table 1:** Search strategy showing meSH/keywords terms used in databases.

No.	Search MeSH/Keywords in PubMed	Results
#1	Biodentine[tw] OR “tricalcium silicate*”[tw] OR “calcium silicate-based*”[tw] OR “root-end filling*”[tw] OR “retrograde filling*”[tw] OR “repair material*”[tw]	4,435
#2	"Enterococcus faecalis"[Mesh] OR “Enterococcus faecalis*”[tw] OR “E. faecalis*”[tw]	20,915
#3	"Anti-Bacterial Agents"[Mesh] OR Antibacterial*[tw] OR antimicrobial*[tw] OR antibiofilm*[tw] OR anti-biofilm*[tw] OR “growth inhibition*”[tw]	712,616
#4	#1 AND #2 AND #3	66
	Search MeSH/Keywords in Scopus	
	TITLE-ABS-KEY(“Biodentine” OR “tricalcium silicate” OR “calcium silicate-based” OR “root-end filling” OR “retrograde filling” OR “repair material”) AND TITLE-ABS-KEY(“Enterococcus faecalis” OR “Enterococcus faecalis” OR “E. faecalis”) AND TITLE-ABS-KEY(“Anti-Bacterial Agents” OR “Antibacterial” OR “antimicrobial” OR “antibiofilm” OR “anti-biofilm” OR “growth inhibition”)	83
	Search MeSH/Keywords in WoS	
	TS = (“Biodentine” OR “tricalcium silicate” OR “calcium silicate-based” OR “root-end filling” OR “retrograde filling” OR “repair material”) AND TS = (“Enterococcus faecalis” OR “Enterococcus faecalis” OR “E. faecalis”) AND TS = (“Anti-Bacterial Agents” OR “Antibacterial” OR “antimicrobial” OR “antibiofilm” OR “anti-biofilm” OR “growth inhibition”)	62

*A most commonly used symbol at the end of the word stem that provides for all variants on the word stem.

### 2.3 Inclusion criteria

This review included all the studies in the English language, conducted *in vivo* on both human and animal subjects as well as *in vitro* on any type of laboratory model.

### 2.4 Exclusion criteria

Studies were excluded based on the following criteria: studies that evaluated the antibacterial activity of Biodentine against bacterial species other than *E. faecalis*; studies that investigated the response of *E. faecalis* to endodontic materials other than Biodentine; studies involving *E. faecalis* mixed with other bacterial species; studies assessing modified versions of Biodentine; studies that examined the dentin/Biodentine interface; and review studies, case reports, and case series.

### 2.5 Study selection and data extraction

In accordance with PRISMA guidelines, two authors (HS and NS) independently screened the included articles and extracted the necessary information. Initially, the title and abstract of each article were assessed and the appropriate studies were retrieved and then thoroughly and carefully examined for eligibility and inclusion in the review. EndNote X8 (Clarivate Analytics, PA, USA) was used to eliminate all duplicated studies and manage the study citations list. In a case of discrepancy between the authors, a discussion was done with a third author (AT) and came to a decision. After screening the included studies, the following data were extracted: authors, year and type of study, type of Biodentine intervention, type of Biodentine samples, *E. faecalis* strain, assessment method, exposure time, main results, and conclusion.

A narrative synthesis was performed to address the diversity in study designs, interventions, and outcomes. The studies were categorized based on their methodologies, type of Biodentine intervention, and the results of the antibacterial activity against *E. faecalis*. The findings were qualitatively summarized, with a focus on identifying shared patterns and notable discoveries across the studies.

### 2.6 Assessment of risk of bias

The quality and risk of bias of the included studies were assessed independently by two authors (HS and SA) in accordance with Modified CONSORT checklist items for pre-clinical *in vitro* studies on dental materials [Figgion et al. (27)]. The criteria included eight domains: intervention, outcomes, sample size calculation, specimen randomization, implementation, operator blinded, statistical analysis, and results (outcomes and estimation). During the assessment process, each domain was reported as YES if the corresponding parameter was explicitly described or NO if the parameter was absent or not fully declared. The third author (AT) resolved the discrepancy between the two authors. The overall risk of bias for each study was determined based on the number of “YES” as: 1–3 refers to high bias; 4–6 refers to medium bias; and 7–8 refers to low bias.

## 3 Results

**Figure 1:**
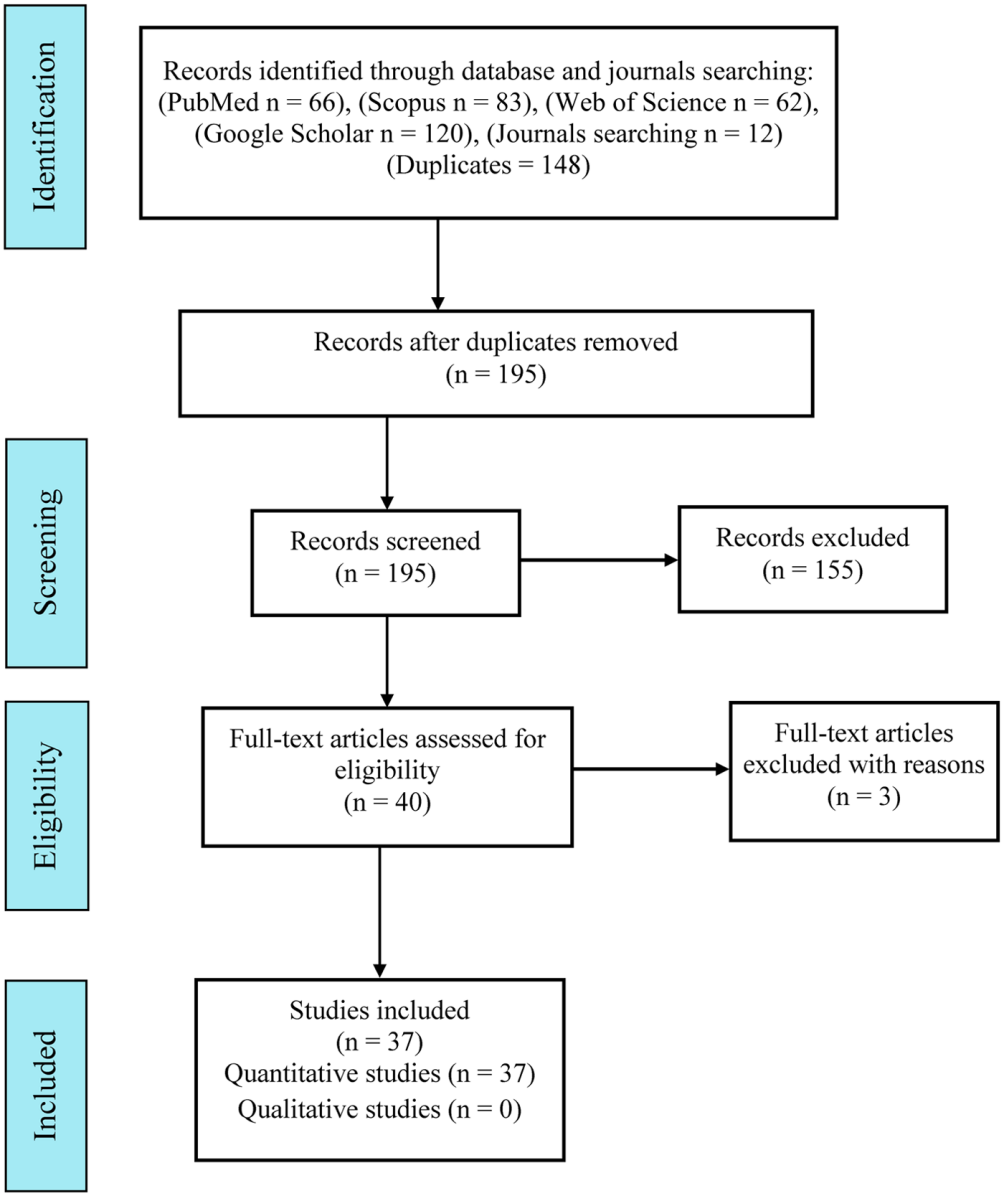
Flow chart showing the strategy used in this review study.

The search in electronic databases retrieved a total of 343 articles [PubMed = 66, Scopus = 83, Web of Science = 62, Google scholar = 120 (top 120 relevant studies), and hand searching in journals = 12]. By the electronic de-duplication, 148 studies were excluded. Then, an independent and comprehensive reading of the titles and abstracts of the remaining 195 articles were performed and 155 articles which did not meet the inclusion criteria were excluded. After retrieving the articles and thoroughly examined them for inclusion and eligibility, three articles were excluded with reasons. Finally, the remaining 37 studies that fulfilled the inclusion criteria were included in this review study. The excluded full-text articles (*n* = 3) were due to the following reasons: *E. faecalis* was mixed with other bacterial species forming dual and multispecies biofilm models (28); A modified Biodentine was used in the antibacterial activity assessment (29); and the absence of a pure Biodentine control (30). The strategy search in the electronic database in this review study has been summarized in Figure 1.

### 3.1 Risk of bias

As illustrated in Table 2, none of the included studies met all the criteria of risk of bias. Of the 37 studies in this systematic review, only 5 studies (13.51%) had a medium risk of bias, whereas the remaining 32 studies (86.48%) showed a high risk of bias. Most of the studies failed or did not clearly describe the sample size calculation, specimen randomization, implementation, and operator blinded parameters.

**Table 2:** Risk of bias of the studies in accordance with modified CONSORT checklist [figgion et al. (27)].

Study	Intervention	Outcomes	Sample size calculation	Specimen randomization	Implementation	Operator blinded	Statistical analysis	Results (outcomes & estimation)	Overall risk of bias
Cruz Hondares et al. (31)	YES	YES	NO	NO	NO	NO	YES	NO	High
Fetouh et al., (32)	YES	YES	NO	NO	NO	NO	YES	NO	High
Ravindran et al. (33)	YES	YES	NO	NO	NO	NO	YES	NO	High
Ravindran et al. (34)	YES	YES	NO	NO	NO	NO	YES	NO	High
Akin et al. (35)	YES	YES	YES	NO	NO	NO	YES	NO	Medium
Ravindran et al. (36)	YES	YES	NO	NO	NO	NO	YES	NO	High
Alkhalidi et al. (37)	YES	YES	NO	NO	NO	NO	YES	NO	High
Al-Yousifany et al. (38)	YES	YES	NO	NO	NO	NO	YES	NO	High
Ashi et al. (39)	YES	YES	NO	NO	NO	NO	YES	NO	High
BAKIR et al. (40)	YES	YES	NO	NO	NO	NO	YES	NO	High
Bhat and Bhagat (41)	NO	YES	NO	NO	NO	NO	YES	NO	High
Bhavana et al. (42)	YES	YES	NO	NO	NO	NO	YES	NO	High
Chopra and Gulve (43)	YES	YES	NO	NO	NO	NO	YES	NO	High
Çırakoğlu et al. (44)	YES	YES	NO	NO	NO	NO	YES	NO	High
Demiryürek et al. (45)	YES	YES	NO	NO	NO	NO	YES	NO	High
Esteki et al. (46)	YES	YES	NO	NO	NO	NO	YES	YES	Medium
Hiremath et al. (47)	YES	YES	YES	NO	NO	NO	YES	NO	Medium
Hiremath et al. (48)	YES	YES	NO	NO	NO	NO	YES	NO	High
Jain et al. (49)	YES	YES	NO	NO	NO	NO	YES	NO	High
Ji et al. (50)	YES	YES	NO	NO	NO	NO	YES	NO	High
Kadam et al. (51)	YES	YES	YES	NO	NO	NO	YES	NO	Medium
Kawle and Saraf (52)	NO	YES	NO	NO	NO	NO	YES	NO	High
Kim et al. (53)	YES	YES	NO	NO	NO	NO	YES	NO	High
Koruyucu et al. (54)	YES	YES	NO	NO	NO	NO	YES	NO	High
Koutroulis et al. (55)	YES	YES	NO	NO	NO	NO	YES	NO	High
Koutroulis et al. (56)	YES	YES	YES	NO	NO	NO	YES	YES	Medium
Naithani et al. (57)	YES	YES	NO	NO	NO	NO	NO	NO	High
Nikhil et al. (58)	YES	YES	NO	NO	NO	NO	YES	NO	High
Nourzadeh et al. (59)	YES	YES	NO	NO	NO	NO	YES	NO	High
Pelepenko et al. (60)	NO	NO	NO	NO	NO	NO	YES	NO	High
Queiroz et al. (61)	YES	YES	NO	NO	NO	NO	YES	NO	High
Sarmamy and Saeed (62)	YES	YES	NO	NO	NO	NO	YES	NO	High
Shalan and Al-Hashimi (63)	YES	YES	NO	NO	NO	NO	YES	NO	High
Varghese et al. (64)	NO	YES	NO	NO	NO	NO	YES	NO	High
Vats and Maheshwari (65)	NO	YES	NO	NO	NO	NO	YES	YES	High
Viswanath et al. (66)	YES	YES	NO	NO	NO	NO	YES	NO	High
Koutroulis et al. (67)	YES	NO	YES	NO	NO	NO	YES	NO	High

### 3.2 General characteristics and assessment methods

The characteristics and details of the included studies are summarized in Table 3. All the 37 studies included in this review article were *in vitro* studies (31–67) and no *in vivo* studies were found. Of these studies, twenty-three studies employed an agar diffusion test (ADT) for assessing the antibacterial activity (32–38, 40–46, 49, 51, 58, 60, 62–66) by measuring the inhibition zone around the test material. Three studies used direct contact test (DCT) to count the colony forming units (CFUs) (39, 61) or optical density (OD) (54). Three studies used antibiofilm assay reading the OD (47, 57) or Log (CFU + 1)/ml (56) of adherent stained biofilm. Two studies used the tube dilution method (48, 52), recording the minimal inhibitory concentration (MIC), and one study used bacterial adhesion assay (55) by a confocal laser scanning microscope to image the viable bacteria. One used the broth dilution method (53) to read the OD. The study by Ji et al. (50) used three methods, ADT, DCT, and carry-over effect test, while Nourzadeh et al. (59), and Koutroulis et al. (67), used the dentine block model to test the antibacterial activity of test materials and CFUs were counted. The study by Cruz Hondares et al. (31), employed two methods, ADT and direct culture test.

**Table 3:** Characteristics of the included studies.

Authors/year/type of study	Type of biodentine intervention (test material or control)	Type of Biodentine samples	*E. faecalis* strain	Assessment method	Exposure time	Main results	Conclusions
Cruz Hondares et al., 2024 (31)/*in vitro*	Biodentine (Septodont, France) compared to ProRoot MTA, MTA Angelus, EndoSequence, NeoMTA 2, and NeoPutty	Discs (4 mm × 2 mm)	ATCC 29212	-Agar diffusion test using bacterial suspension of 1 × 10^8^ CFU/ml (100 µl) and Todd Hewitt agar containing 0.5% yeast extract (THY). -Direct culture test using 100 µl bacterial suspension of 11 × 10^7^ CFU/ml in 1 ml of THY medium containing test materials and cultured on THY agar	-Agar diffusion test at 48 h -Direct contact test at 48 h	-Biodentine had no effect against *E. faecalis* with a mean of inhibition zone of 0.00 (±0.0) by agar diffusion test. -Biodentine showed significant inhibitory effects against *E. faecalis* (*p* < .001) in direct culture test.	All the tested materials have acceptable *in vitro* antimicrobial, biocompatible, and mineralization-supporting properties, with Biodentine demonstrating more favourable *in vitro* antimicrobial activity among the tested materials.
Fetouh et al., 2024 (32)/*in vitro*	Biodentine (Septodont, France) compared to Dycal and Rootdent MTA	Cavities (10 mm × 2 mm) were punched in the agar and instantly filled with the test materials.	N/A	Agar diffusion test using bacterial suspension of approximately 10^8^ CFU/ml (0.5 McFarland) in Muller Hinton (MH) broth and MH agar	24 h, 48 h, and 7 days	Biodentine exhibited a mean of inhibition zones of 6.89 mm (±0.53), 7.73 mm (±0.51), and 6.27 mm (±0.28) for day 1, 2, and 7, respectively. Biodentine had greater antibacterial activity than Dycal and lower than Rootdent MTA against *E. faecalis.*	All tested materials are effective in inhibiting the growth of both microorganisms (*S. mutans* and *E. faecalis*). Rootdent MTA was more effective against *E. faecalis*, while Biodentine was more effective against *S. mutans*.
Ravindran et al., 2024 (33)/*in vitro*	Biodentine (Septodont, France) as a control compared to MTA Angelus, MTA formulation with Metronidazole in powder, and MTA formulation with Metronidazole in liquid	Wells (4 mm × 5 mm) were prepared in agar plate and filled with freshly mixed test materials	ATCC 29212	Agar diffusion test using Mueller-Hinton agar	24 h	Biodentine had a mean of inhibition zones of 19.45 mm (±0.26) compared to 14.33 mm (±0.45) for MTA Angelus and 23.54 mm (±0.86) for MTA formulation with Metronidazole in powder, and 23.76 mm (±0.28) for MTA formulation with Metronidazole in liquid. The difference was found to be statistically significant (*p* < 0.05).	-The conventional MTA and Biodentine showed some amount of antimicrobial activity. -The results showed that the addition of metronidazole, either in the liquid or powder component, can have additive effects on the antimicrobial properties of the modified MTA
Ravindran et al., 2023 (34)/*in vitro*	Biodentine (Septodont, France) as a control compared to MTA Angelus, MTA formulation with Doxycycline in powder, and MTA formulation with Doxycycline in liquid	Wells (4 mm × 5 mm) were prepared in agar plate and filled with freshly mixed test materials	ATCC 29212	Agar diffusion test using Mueller-Hinton agar	24 h	Biodentine had a mean of inhibition zones of 21.35 mm (±0.60) compared to 15.03 mm (±0.29) for MTA Angelus and 25.81 mm (±0.40) for MTA formulation with Metronidazole in powder, and 25.01 mm (±0.18) for MTA formulation with Metronidazole in liquid. The difference was found to be statistically significant (*p* < 0.05)	-The conventional MTA and Biodentine showed some amount of antimicrobial activity. -The addition of doxycycline, either in the liquid or powder component, can have additive effects on the antimicrobial properties of the modified MTA
Akin et al., 2023 (35)/*in vitro*	Biodentine (Septodont, France) compared to ProRoot MTA, TheraCal LC, and Dycal after complete setting reactions (21 days)	Discs-shaped specimens (8 mm × 2 mm)	ATCC 29212	Agar diffusion test using bacterial suspension of approximately 5.8 × 10^6^ cfu/ml and Tyriptic Soy Agar	24 h, 48 h, and 72 h	-Biodentine exhibited a mean of inhibition zones of 0.0 mm against *E. faecalis* at day 1, 2, and 3. -A limited diffusion was observed against *E. faecalis* in Biodentine group, but this did not result with inhibition zone.	The tested pulp-capping materials did not represent antibacterial activity after the completed setting reaction, except a limited zone of inhibition against *S. mutans* in Dycal group.
Ravindran et al., 2023 (36)/*in vitro*	Biodentine (Septodont, France) as a control compared to MTA Angelus, and newly modified MTA	Wells (5 mm × 4 mm) were prepared in agar and filled with the freshly mixed test materials	ATCC 29212	Agar diffusion test using Mueller-Hinton agar	24 h	Biodentine exhibited a mean of inhibition zone of 20.67 mm (±0.98) against *E. faecalis* compared to 13.33 mm (±0.58) for MTA Angelus and 24.67 mm (±0.78) for the modified MTA. There were statistically significant differences (*p* < .01) among the tested materials.	-All tested materials showed a considerable amount of antimicrobial activity against *E. faecalis*, *S. mutans*, and *C. albicans*. -The newly modified MTA could serve as an alternative to the conventional MTA in terms of faster setting, higher strength, and better antimicrobial properties.
Alkhalidi et al., 2015 (37)/*in vitro*	Biodentine (Septodont, France) as a control to compare with a newly prepared calcium based cement and Zinc polycarboxylate cement (control)	Discs (6 mm × 2 mm)	Clinical isolate	Agar diffusion test using bacterial suspension of approximately 1.5 × 10^8^ organisms/ml (McFarland 0.5 in BHI broth) and Muller–Hinton agar	24 h	Biodentine exhibited a mean of inhibition zone of 2.9 mm (±0.567) against *E. faecalis* (Size of inhibition zone = diameter of halo - diameter of the disc). There were statistically significant differences (*p* < 0.0001) among the inhibition zones produced by the tested materials	It was concluded that new calcium based cement has a better antimicrobial properties than Biodentine and polycarboxylate cement
Al-Yousifany et al., 2015 (38)/*in vitro*	Biodentine (Septodont, France) as a control to compare with a newly prepared calcium based cement	Discs (6 mm × 1 mm)	N/A	Agar diffusion test using bacterial suspension of approximately 1.5 × 10^8^ organisms/ml (McFarland 0.5 turbidity in PBS)	24 h	Biodentine had a mean of inhibition zone of 9.25 mm (±0.26) against *E. faecalis*, with statistically lower antibacterial effect compared to the experimental cement	All microbial species were inhibited by the two types of materials used in the currents study with different levels
Ashi et al., 2022 (39)/*in vitro*	Biodentine [Septodont, France (Lot: B28033)] compared to MTA Biorep and Well-Root PT.	One milliliter of the bacterial medium was put to each well of 24-well culture plates that contain material sample.	ATCC 29212	Direct contact test using bacterial suspension of a turbidity of OD_600_ (nm) = 0.3 in BHI broth and BHI agar plate	24 h	Bacterial growth was significantly inhibited with the three cements. No significant difference was found among them for the efficiency against *E. faecalis* (*p* > 0.05). The three cements killed about 50% of the bacteria after 24 h vs. the control (*p* < 0.05)	The three CSC products, MTA Biorep, Biodentine and Well-Root PT, had a high antibacterial activity, formation of phosphate crystal in PBS alkaline and had comparable solubility. The premixed format was more convenient as a retrograde agent.
BAKIR et al., 2021 (40)/*in vitro*	Biodentine [Septodont, France (Lot: B22596)] compared to TheraCal LC, Dycal, Calcimol LC, Activa and MTA Angelus.	Discs (5 mm × 2 mm)	ATCC 29212	Agar diffusion test using bacterial suspension of approximately 1.5 × 10^8^ CFU/ml [0.5 McFarland turbidity in physiological salt solution (PSS)] and BHI agar	24 h, 48 h	Biodentine exhibited mean of inhibition zones of 6.6 mm (±0.22) and 6.7 mm (±0.26) at 24 h and 48 h, respectively against *E. faecalis*. All test materials were significantly effective. Biodentine had no significant difference with Calcimol LC and Activa at the end of 24 h and 48 h, however it was less effective (*p* < 0.05) compared to other materials.	The antibacterial effect of pulp capping agents against *S. mutans L. acidophilus E. faecalis bacteria*, which is involved in the formation and development mechanism of caries, contributes to the preservation of pulp vitality
Bhat and Bhagat, 2019 (41)/*in vitro*	Biodentine compared to MTA	Fifty milligram of the material was prepared and filled into 4 mm-wells in the agar plates	N/A	Agar diffusion test using BHI broth	24 h	It was found that the zone of inhibition against *E. faecalis* was 3.4 mm with biodentine material. The difference was non-significant (*P* > 0.05) with MTA	Authors found both materials effective against *S. mutans* and *E. faecalis*. However, Biodentine produced higher inhibition zone than MTA
Bhavana et al., 2015 (42)/*in vitro*	Biodentine (Septodont, France) compared to GIC and ProRoot MTA	Wells (4 mm × 4 mm) were prepared on plates and filled with freshly manipulated test materials	ATCC 29212	Agar diffusion method using bacterial suspension of approximately 5 × 10^8^ CFU/ml in trypticase soy broth and Meuller-Hinton agar plates	24 h	Biodentine showed a mean of inhibition zone of 3.1 mm against *E. faecalis*. The antimicrobial activity of Biodentine, on all the microorganisms tested, was very strong, showing a mean inhibition zone of 3.2 mm, which extends over time towards all the strains	All materials showed antimicrobial activity against the tested strains except for GIC on Candida. Biodentine created larger inhibition zones than MTA and GIC
Chopra and Gulve, 2016 (43)/*in vitro*	Biodentine (Septodent, France) compared to MTA Angelus and RMGIC	Wells (7 mm diameter) were prepared in agar and immediately filled with the test materials	ATCC 29212	Agar diffusion test using bacterial suspension of approximately 5 × 10^6^ CFU/ml in Tripticase Soy Broth and Mueller Hinton agar	24 h	Biodentine exhibited a mean of inhibition zone of 14.66 mm (±0.57). Inhibition zones for Biodentine against *E.faecalis* and *C.albicans* were significantly larger (*P* < 0.01) when compared to MTA and RMGIC.	All materials showed antimicrobial activity against the tested strains except RMGIC against *C.albicans*. Biodentine had a greater antimicrobial activity compared to MTA and RMGIC.
Çırakoğlu et al., 2020 (44)/*in vitro*	Biodentine (Septodont, France) compared to MTA Repair HP, NeoMTA Plus, ProRoot MTA and MTA Angelus	Wells (5 mm × 3 mm) were punched in agar, and filled with the test materials	ATCC 29212	Agar diffusion method using bacterial suspension of 0.5 McFarland turbidity standard in PPS and Mueller-Hinton agar plates. Inhibition zone = (diameter of halo - diameter of the specimen) × 1/2	24 h, 48 h	-Biodentine revealed mean of inhibition zones of 0 and 8.66 mm (±1.25) after 24 and 48 h, respectively against *E. faecalis*. -No inhibitory activity was exhibited by any of the tested materials against *E. faecalis* within 24 h. At the end of the 48-h incubation period, a slight inhibition was detected by all of the five tested materials against *E. faecalis*	Although there was no clear indication of inhibitory activity of the materials tested against *E. faecalis* at 24 h, slight antibacterial activity was detected after 48 h
Demiryürek et al., 2016 (45)/*in vitro*	Biodentine (Septodont, France) compared to MTA Angelus and DiaRoot BioAggregate	Pits (5 mm × 2 mm) were formed on agar and the test materials were prepared and filled into the open pits	ATCC 29212	Agar diffusion method using bacterial suspension of 0.5 McFarland standard in Brucella broth medium and planted on Mueller Hinton agar	12 h, 24 h, 48 h, 72 h	Biodentine exhibited mean of inhibition zones of 13.21 mm (± 0.18), 13.32 mm (± 0.24), 13.12 mm (± 0.13), and 10.22 mm (± 0.13) at 12 h, 24 h, 48 h and 72 h, respectively against *E. faecalis*. Antimicrobial activity of Biodentine vs. *E. faecalis* was statistically higher than MTA Angelus and DiaRoot BioAggregate (*P* < 0.001).	Within the limits of our study, retrograde filling materials MTA Angelus, Biodentine and DiaRoot BioAggregate exhibited antibacterial and antifungal effect
Esteki et al., 2021 (46)/*in vitro*	Biodentine (Septodont, France) compared to MTA and calcium-enriched mixture (CEM)	Wells (5 mm × 4 mm) were prepared on agar plate and immediately filled with freshly mixed materials	ATCC 29212	Agar diffusion method using bacterial suspension of 1.5 × 10^8^ CFUs/ml (0.5 McFarland in trypticase soy broth and Sabouraud dextrose broth) and Mueller-Hinton agar plates	24 h	Biodentine exhibited a mean of inhibition zone of 9.46 mm (±1.06) against *E. faecalis*. The inhibitory effect of Biodentine on *E. faecalis* and C. albicans was significantly superior to that of the other two cements (*P* < 0.05)	All cements revealed antimicrobial properties against the tested microbial strains. Biodentine had stronger antimicrobial effects against *E. faecalis* and C. albicans compared to MTA and CEM cement
Hiremath et al., 2020 (47)/*in vitro*	Biodentine (Septodont, France) compared to MTA Angelus and MTA plus with/without conjugated with chitosan	Test prepared materials (with/without chitosan) were serially two-fold diluted with PBS	ATCC 29212	Antibiofilm efficacy using crystal violet stain of 3-day biofilm that grown and cultured in BHI broth and Trypticase soy broth culture	24 h	The OD scores for Biodentine was (0.28 ± 0.21) against *E. faecalis*. However, there was no significant difference seen with Biodentine-chitosan conjugate (0.20 ± 0.10). Generally, there was a mean clinical reduction in the biofilms of the conjugates as compared to their individual counter parts	Within the limitations of the present study, all the materials proved to have antibiofilm action against *E. faecalis*
Hiremath et al., 2015 (48)/*in vitro*	Biodentine (Septodont, France) compared to ProRoot MTA and MTA Plus	Doubling dilutions of the materials were prepared (10 mg/ml - 0.156 mg/ml)	N/A	Tube dilution method using BHI broth for dilution and Mac Conkey's agar for subculture	24 h, 48 h, 72 h	The results showed that Biodentine and ProRoot MTA inhibited the growth of majority of strains of *E. faecalis*; whereas, MTA Plus was not that effective. The MIC at which Biodentine and ProRoot MTA were effective in inhibiting *E. faecalis* is 5 mg/ml	Biodentine and ProRoot MTA proved to have antimicrobial property
Jain et al., 2018 (49)/*in vitro*	Biodentine (Septodont, France) compared to ProRoot MTA	Wells (4 mm × 4 mm) were prepared on agar and immediately filled with manipulated test materials	N/A	Agar diffusion method using BHI broth and Mueller-Hinton agar plates	24 h	Biodentine exhibited a zone of inhibition of 3.1 mm against *E. faecalis*. Inhibition zones formed by Biodentine against *E. faecalis* and *S. mutans* were significantly larger than the zones formed by MTA (*P* < 0.05)	It can be concluded that Biodentine and MTA have antimicrobial activity against *E. faecalis* and *S. mutans*, but higher mean zone of inhibition was recorded in Biodentine
Ji et al., 2022 (50)/*in vitro*	Biodentine (Septodont, France) compared to ProRoot MTA and iRoot FS	-In ADT, filter papers (5 mm diameter) coated with test materials were placed on each agar plate. -Filter papers coated with test materials were placed with 200 µl of the bacteria suspension in DCT, and with saline (20 µl), broth (230 µl) and diluted with broth and bacterial suspension in Carry-over effect test.	ATCC 19433	-Three sets of test materials were assessed (20 min, 1 day, and 7 days after mixing) -Agar diffusion test with bacterial suspension of 10^8^ CFU/ml (turbidity of 0.1 OD) and streaked on BHI agar plates. - DCT using bacterial suspension (10^7^ CFU/ml) in direct contact with filter paper coated with the materials and cultured on BHI agar. - Carry-over effect test using bacteria suspension (1.5 × 10^8^ CFU/ml) and BHI agar plates	-Agar diffusion test at 48 h - DCT after 1 h - Carry-over effect test at 48 h	-No inhibition zone was observed in ADT -All three materials presented highest antimicrobial effect against *E. faecalis* and P. gingivalis when freshly mixed. -Biodentine inhibited most *E. faecalis* (80.7%) and had higher antimicrobial activity than iRoot FS after 1 and 7 days. -Carry-over of the antimicrobial effect from the materials was not observed	Fresh Biodentine, iRoot FS, and MTA killed *E. faecalis* and P. gingivalis effectively, but their antimicrobial effect decreased after 24 h, and distinctly decreased after 7 days after mixing.
Kadam et al., 2020 (51)/*in vitro*	Biodentine compared to MTA and EndoSequence	wells (4 mm × 4 mm) were made on agar and filled with freshly mixed cements	ATCC 29212	Agar diffusion method using bacterial suspension of 5 × 10^6^ CFU/ml in Trypticase Soy Broth and Muller Hinton Agar.	24 h, 48 h, 72 h	-Biodentine demonstrated mean of inhibition zones of 5.5 mm (±0.96), 6.0 mm (±0.98) and 6.0 mm (±0.98) at 24 h, 48 h and 72 h, respectively. EndoSequence showed the highest antimicrobial efficacy against *E. faecalis* compared to that of Biodentine and MTA (*p* < 0.05)	-All the three cements showed antimicrobial activity against *E. faecalis*. Compared to Biodentine and MTA, EndoSequence showed the largest zone of inhibition
Kawle and Saraf, 2020 (52)/*in vitro*	Biodentine compared to MTA Angelus and Theracal LC	N/A	N/A	Tube dilution method using BHI broth and Subcultured on Mac Conkey's agar	24 h	Biodentine demonstrated a mean of CFU counts of *E. faecalis* of 59.27 (±29.76). The MIC was highest and CFU's were lowest with Biodentine in comparison with other two materials	Biodentine was found to be more anti-bacterial as compared to MTA angelus and theracal LC
Kim et al., 2023 (53)/*in vitro*	Biodentine™ (Septodont, France) compared to Theracal PT® and Theracal LC®	Materials extracts with concentration of 200 mg/ml and two-fold serial dilutions in saline	ATCC 29212	According to the method given by the Clinical Laboratory Standard Institute (68) using bacterial suspension of 1.0 × 10^6^ cells/ml in BHI broth.	24 h	-A direct correlation was found between all test materials and their concentrations (*p* < 0.05). -The sequence of antibacterial effects were Theracal PT, Theracal LC, Biodentine, and control groups	The study demonstrated the high antibacterial activity of Theracal PT and the low antibacterial effect of Biodentine
Koruyucu et al., 2015 (54)/*in vitro*	Biodentine (Septodont, France) compared to MTA Angelus and Dycal	The prepared materials were set in wells of 96-well plates and 10 µl of bacterial suspension placed directly on them and BHI broth were added	ATCC 29212	Direct contact test of 3 sets of test materials (within 20 min of recommended setting time, 24-h and 1-week) using bacterial suspension of a concentration of 10^6^ CFU/ml in BHI broth	1-h intervals among 24 h	-Biodentine exhibited scores of 0.87 ± 0.65, 0.98 ± 0.55 and 0.87 ± 0.56 for 20-min, 1 day and 1 week, respectively. -The antibacterial activity of Biodentine remained at the same standard levels in a week period although antibacterial activity of MTA getting decreased and had the same levels with Biodentine. -MTA and Biodentine samples showed significant differences with Dycal	While freshly mixed MTA showed the best antibacterial activity over time, Biodentine had shown similar antibacterial activity to MTA
Koutroulis et al., 2022 (55)/*in vitro*	Biodentine (Septodont, France) as a reference material compared to TZ-base, TZ-bg10, TZ-bg20, TZ-Ag0.5, TZ-Ag1, TZ-Ag2, and IRM (reference material)	Material specimens (9 mm diameter) exposed to 700 µl of bacterial suspension (materials aged at 1, 7, and 28 days in water or FBS)	N/A	Bacterial adhesion assay by fluorescence microscopy using bacterial inoculum of 1 × 10^8^ CFU/ml (OD = 1) in PBS	1 h	In Biodentine, all samples allowed *E. faecalis* adhesion. However, bacterial adhesion was significantly lower than the positive control for the 1- and 7-day samples (*p* < 0.05). No significant differences were reported between media for the same evaluation periods (*p* > 0.05)	All 28-day aged materials failed to inhibit bacterial adherence. The measured physical parameters did not appear to be related to the degree of bacterial adhesion
Koutroulis et al., 2023 (56)/*in vitro*	Biodentine (Septodont, France) compared to TZ-base, TZ-bg10, TZ-bg20, TZ-Ag0.5, TZ-Ag1, TZ-Ag2, and IRM	Material leachates was prepared by immersing materials discs (9 mm × 1 mm) in 4 ml ultrapure water or FBS and aged for 1, 7, and 28 days or samples were prepared inside cell culture inserts and placed immediately in 1.8 ml medium for 24 h.	ATCC 47077	Antibiofilm assay using bacteria inocula of 10^8^ CFU/ml in Tryptic Soy Broth (TSB) and plated on TSB agar plates	24 h	-Biodentine and 20% bioactive glass-containing cement had overall lower alkalinity, calcium release, and antibacterial activity than TZ-base, and Biodentine was less cytotoxic than TZ-base. -FBS leachates showed reduced antibacterial activity compared to water leachates	-Biodentine showed reduced antibacterial capacity and cytotoxicity than TZ-base. -It can be concluded that exposure conditions (immersion medium and aging period) significantly affected the materials’ leaching properties
Naithani et al., 2022 (57)/*in vitro*	Biodentine compared to MTA and MTA Plus	Serial two-fold dilutions of combinations were prepared in PBS	ATCC 29212	Antibiofilm assessment using BHI broth and Trypticase soy broth culture	24 h	Biodentine demonstrated a mean OD of 0.29 compared to 0.23 for MTA Plus and 0.18 for MTA. The difference was non- significant (*P* > 0.05)	All the materials proved to have antibiofilm action against *E. faecalis*
Nikhil et al., 2014 (58)/*in vitro*	Biodentine was tested as a control to compare with Biodentine/chlorhexidine and Biodentine/doxycycline	Wells (4 mm × 4 mm) were prepared on the medium and filled with the freshly prepared test materials.	ATCC 29212	Agar diffusion method using bacterial suspension of 0.5 McFarland standard in BHI broth and inoculated on blood agar plates	24 h, 48 h, 72 h	-Biodentine demonstrated mean of inhibition zones of 7.5 ± 0.5 mm, 7.5 ± 0.5 mm and 7.5 ± 0.5 mm at 24 h, 48 h and 72 h, respectively against *E. faecalis*. -All Biodentine samples inhibited microbial growth. The highest means of inhibition zone for all the micro-organisms were found around Biodentine/chlorhexidine (13.417) followed by Biodentine alone (12.236) and Biodentine/doxycycline (11.25).	-The antimicrobial activity of Biodentine was clear against tested bacteria and fungi. -Addition of 2% chlorhexidine to Biodentine enhanced its antibacterial activity, while addition of 10% doxycycline to Biodentine decreased its antibacterial activity
Nourzadeh et al., 2019 (59)/*in vitro*	Biodentine (Septodont, France) compare to calcium-enriched mixture (CEM)	The canals of root segments (7-mm in length) prepared from extracted teeth were filled with the prepared test materials	ATCC 29212	The canals of root segments made from extracted teeth were prepared and filled with bacterial suspension of 0.5 McFarland (1.5 × 10^8^ CFU/ml) in BHI. The canals were cleaned and filled with test materials. 1-mm hole was drilled into the root and the shavings fell into test tubes containing BHI and CFUs were counted	7 days, 30 days	-Biodentine had mean CFU levels of 3.205 (±0.202) and 2.35 (±0.271) at 7 days and 30 days respectively. -Compared to positive control, Biodentine decreased the number of bacteria at both time intervals, but a statistical significance was seen only after 30 days (*P* < 0.01)	Although both biomaterials exerted antibacterial activity against *E. faecalis*, the CEM cement had more antibacterial activity than Biodentine
Pelepenko et al., 2021 (60)/*in vitro*	Biodentine (Batch#B22869) compared to White-MTA Flow and ProRoot MTA	In direct contact test, 0.10 g of set materials were used in BHI broth	ATCC 29212	Agar diffusion method and direct contact test	Direct contact test at 3 h, 24 h, 48 h	-No inhibition halos were observed for the cement, regardless of the bacteria tested in the agar diffusion test. -Direct contact with planktonic *E. faecalis* showed significantly higher values of turbidity and an apparent upregulation of the bacterial growth for all the tested materials	None of the materials exhibited inhibition halos against the tested bacteria, and similar turbidity values were obtained after 48 h in direct contact with *E. faecalis*, indicating an upregulation to bacterial growth
Queiroz et al., 2021 (61)/*in vitro*	Biodentine (Septodont, France) compared to TCS, TCS ZrO_2_ + 10% Biosilicate, and TCS ZrO_2_ + 20% Biosilicate	0,2 µl of prepared test materials inserted into wells of microplates before the bacterial suspension deposited on it	N/A	Direct contact test using bacterial suspension of 10^8^ CFU/ml in BHI broth and seeded on Tryptic soy agar	48 h	-Biodentine had a mean CFU ml^−1^log_10_ of 0.0 (±0.0) against *E. faecalis*. All materials presented antibacterial activity, differing from the control group (*p* < .05). Only Biodentine and the association of 20% Biosilicate to TCS with ZrO2 were able to completely eliminate the number of bacteria	These experimental cements demonstrated antimicrobial activity and mineralization nodules formation, suggesting their potential for clinical use
Sarmamy and Saeed, 2020 (62)/*in vitro*	Biodentine (Septodont, France) as a control material compared to an experimental cement and MTA (control)	Disc-shaped specimens (6 mm × 2 mm) were prepared and placed on agar	Clinical isolate	Agar diffusion test using bacterial suspension of 1.5 × 10^8^ organisms/ml (McFarland 0.5 turbidity in BHI broth) and innoculated on Muller-Hinton agar plates. Size of inhibition zone = diameter of halo – diameter of the disc	24 h	-Biodentine exhibited a mean of inhibition zone of 9.2 mm (±1.68). -All materials showed antibacterial activity	All tested materials have antibacterial properties against the tested bacteria. The cement-based capping material prepared from egg shells and the biopolymer chitosan has better antimicrobial properties than Biodentine and MTA
Shalan and Al-Hashimi, 2015 (63)/*in vitro*	Biodentine (ZiZine, France) compared to ProRoot MTA with/without aqueous solutions of black seed extract	Cavities (5 mm × 4 mm) were made in each agar plate and filled with the prepared test materials	N/A	Agar diffusion method using bacterial suspension of 0.5 McFarland standards and inoculate on Muller Hinton agar	24 h	-Biodentine had a mean of inhibition zone of 4.02 mm (±0.109). -The antimicrobial action of Biodentine was superior to that of MTA. Biodentine showed a remarkable antibacterial effect, which was increased with the addition of the aqueous solutions of black seed extract	It could be concluded that Biodentine, as well as, MTA are promising materials since they have the potential to inhibit the growth of *E. faecalis*. Moreover, the adding aqueous solutions of black seed extract increased their antibacterial activity against *E. faecalis*
Varghese et al., 2022 (64)/*in vitro*	Biodentine (Septodont, France) compared to MTA Angelus and a new calcium silicate material	Wells (4 mm × 4 mm) were prepared on plates and filled with the freshly manipulated test materials	N/A	Agar diffusion method	24 h	Biodentine demonstrated a mean of inhibition zone of 10.0 mm (±1.5) against *E. faecalis*. The new calcium silicate material shows significantly better antibacterial properties when compared to the other two materials	All three materials showed bacterial inhibition. The new calcium silicate material showed the best results when compared to MTA and Biodentin against *S. mutans* and *E. faecalis*
Vats and Maheshwari, 2019 (65)/*in vitro*	Biodentine (Septodont, France) compared to MTA Angelus	4-mm wells were prepared on agar plates for test materials	N/A	Agar diffusion method using brain heart infusion broth	24 h	Biodentine had a mean of inhibition zone of 3.2 mm against *E. faecalis*. The difference was non-significant (*P* > 0.05) with MTA	Authors found both the tested materials effective against *S. mutans* and *E. faecalis*. However, Biodentine produced higher inhibition zone than MTA
Viswanath et al., 2021 (66)/*in vitro*	Biodentine (Septodont, France) compared to MTA Plus and Endosequence root repair material (ERRM)	Pits (5 mm × 2 mm) were prepared on agar plate and filled with the prepared materials	ATCC 29212	Agar diffusion method using bacterial suspension of 0.5 McFarland turbidity in BHI broth and inoculated on Mueller Hinton agar	24 h, 48 h, 72 h	-Biodentine exhibited mean of inhibition zones of 16 mm (±0.816), 16 mm (±1.155), and 15.57 mm (±0.787) at 24, 48, and 72 h, respectively against *E. faecalis*. -Results showed that all three groups showed significant antimicrobial and antifungal activity (*p* < 0.05). The antimicrobial activity of Biodentine against *E. faecalis* was statistically higher than MTA Plus and ERRM	Biodentine exhibited the greatest antimicrobial activity and MTA Plus exhibited the greatest antifungal activity among the three groups.
Koutroulis et al., 2023 (67)/*in vitro*	Biodentine (Septodont, France) compared to TZ-base, TZ-bg20, TZ-bg40, TZ-Ag1, TZ-Ag2, TotalFill (TF) and IRM	The canals of root dentin segments (3-mm length) prepared from extracted teeth were filled with test materials	ATCC 47077	Split-tooth model using root segments, which were prepared and filled with materials. Three-day biofilms of *E. faecalis* [prepared by adding 2 µl bacterial inoculum (10^8^ CFUs/ml) on membrane filter] were placed in contact with the material. The segments with biofilms were immersed in PBS, vortexed, serially diluted, and plated. Then, CFUs were counted.	1 days, 28 days	The results showed that all one-day material surfaces were antibacterial, and the bactericidal effect against *E. faecalis* was reduced between the 1 and 28 days samples (*p* < 0.05). No statistical differences were found among Biodentine, TF, and IRM at days 1 and 28.	It was concluded that all one-day material and dentin surfaces were antibacterial. TZ-base, TZ-Ag1 and TZ-Ag2 caused a higher logCFU-reduction in *E. faecalis* biofilms than that of the commercial cements in the 28-day test period (*p* < 0.01).

### 3.3 Biodentine intervention in the included studies

Twenty-three studies investigated the antibacterial activity of Biodentine compared to other commercial endodontic materials such as MTA Biorep, Well-Root PT, TheraCal LC, Theracal PT, Dycal, Calcimol LC, Activa, iRoot FS, Endosequence root repair material (ERRM), ProRoot MTA, MTA Angelus, MTA, MTA Repair HP, NeoMTA Plus, NeoMTA 2, NeoPutty, White-MTA Flow, MTA Plus, GIC, RMGIC, Rootdent MTA, DiaRoot BioAggregate and Calcium-enriched mixture (CEM) (31, 32, 35, 39–46, 48–54, 57, 59, 60, 65, 66), whereas fourteen studies investigated the antibacterial activity of Biodentine as a control/reference material compared to experimental materials (33, 34, 36–38, 47, 55, 56, 58, 61–64, 67).

### 3.4 Effectiveness of biodentine against *E. faecalis*

Of the thirty-seven studies included in this review article, thirty studies reported a good antibacterial activity of Biodentine against *E. faecalis* (32–34, 36, 38–43, 45–49, 51–59, 61–66), one study reported a limited antibacterial activity of Biodentine (37), four studies reported a conflicting antibacterial activity, whereas Ji et al. (50) study exhibited no bacterial inhibition for Biodentine using ADT and carry-over effect test while a good antibacterial activity was shown using DCT, Cruz Hondares et al. (31) found no antibacterial activity using ADT, while a significant inhibitory effect was detected using direct culture test, a study by Çırakoğlu et al. (44) revealed no bacterial inhibition at 24 h while a limited antibacterial activity was found at 48 h, and a study by Koutroulis et al. (67) revealed antibacterial activity at 24 h, while no bacterial count reduction was observed at 28 days. Only two studies reported negative antibacterial activity of Biodentine against *E. faecalis* using both ADT and DCT (60) and ADT (35).

The antibacterial activity of Biodentine at various time intervals has been conducted in thirteen studies (32, 35, 40, 44, 45, 48, 51, 54, 58–60, 66, 67). In three studies, there was no statistically significant difference (*p* > 0.05) in the antibacterial activity of Biodentine against *E. faecalis* at different time intervals (40, 48, 66). Other studies reported an increased antibacterial activity (*P* < 0.01) at 30 days compared to 7 days (59), and at 48 h compared to 24 h (44). However, a reduction was reported in two studies at 72 h compared to 12, 24, and 48 h (*P* < 0.001) (45), and in 24 h compared to 28 days (*p* < 0.05) (67). One study (32) reported increased antibacterial activity at 48 h compared to 24 h, followed by a decrease at day 7 (*p* = .012). The level of Biodentine's antibacterial activity remained nearly in same level across three other studies (51, 54, 58), while two studies reported no antibacterial activity (35, 60).

The antibacterial activity of different time interval-aged Biodentine against *E. faecalis* has been shown in two studies (55, 56). A study by Koutroulis et al. (55) revealed no significant difference (*p* > 0.05) in the antibacterial activity of Biodentine aged for 1, 7, and 28 days in water or FBS. While, a study by Koutroulis et al. (56) found that bacterial inhibition in water leachates increased over time when the medium was not refreshed (days 7–28) but decreased when the medium was changed. However, FBS leachates demonstrated lower antibacterial activity compared to water leachates.

### 3.5 The antibacterial activity of biodentine in comparison to commercial endodontic materials

Eleven studies investigated the antibacterial efficacy of Biodentine compared to MTA against *E. faecalis* (31, 35, 41, 42, 48–51, 57, 62, 63). The studies reported a significant (*p* < 0.05) superiority of Biodentine in killing *E. faecalis* in 3 studies (48, 49, 63), and non-significantly (*p* > 0.05) in 5 studies (31, 41, 42, 50, 51), which indicates a clinical reduction in bacterial growth. Whereas, Biodentine was inferior in bacterial inhibition compared to MTA in 2 studies, significantly (*p* < 0.05) in one study (62), and non-significantly (*p* > 0.05) in one study (57). A study by Akin et al. (35) demonstrated no antibacterial efficacy for Biodentine and MTA after a complete setting reaction.

The comparison of Biodentine and MTA Angelus against *E. faecalis* appeared in 13 studies. Biodentine exhibited significantly (*p* < 0.05) greater antibacterial activity in 8 studies (31, 33, 34, 36, 43, 45, 46, 52) and non-significantly (*p* > 0.05) in 2 studies (64, 65), whereas MTA Angelus exhibited significantly (*p* < 0.05) greater antibacterial activity in one study (40) and non-significantly (*p* > 0.05) in 1 study (47). One study (54) reported lower (*p* > 0.05) antibacterial activity for Biodentine at immediate time point and day 1, and higher (*p* > 0.05) activity at 1 week compared to MTA Angelus.

Four studies investigated the effectiveness of MTA Plus and Biodentine against *E. faecalis* (47, 48, 57, 66). Of these studies, two studies (48, 66) reported better antibacterial activity (*p* < 0.05) for Biodentine compared to MTA Plus. Whereas, two studies (47, 57) showed that MTA Plus was superior to Biodentine with no significant difference (*p* > 0.05). Two studies evaluated the effectiveness of CEM in comparison to Biodentine against *E. faecalis* (46, 59). One study (46) reported significantly (*p* < 0.05) better antibacterial activity for Biodentine, and one study (59) reported that Biodentine was significantly (*p* < 0.05) inferior compared to CEM. In addition, of the 3 studies investigated ERRM in comparison to Biodentine against *E. faecalis* (31, 51, 66). Biodentine exhibited significantly (*p* < 0.05) better antibacterial activity in one study (66), and non-significantly (*p* > 0.05) in one study (31); whereas, one study (51) reported that ERRM had significantly (*p* < 0.05) better antibacterial activity than Biodentine.

The antibacterial activity of Biodentine compared to IRM was reported in three studies (55, 56, 67). In two studies (55, 67), Biodentine demonstrated antibacterial activity similar (*p* > 0.05) to that of IRM. In the other study (56), Biodentine exhibited statistically higher antibacterial activity in leachates prepared over 28 days without a medium change, similar activity to IRM in leachates prepared over 28 days with weekly medium changes, and lower antibacterial activity in leachates prepared in the cell culture insert (0–24 h). One study by Koutroulis et al. (67), compared the antibacterial activity of Biodentine and TotalFill, finding that both exhibited similar (*p* > 0.05) antibacterial effects at 1 and 28 days.

Four studies evaluated TheraCal LC against *E. faecalis* and compared to Biodentine (35, 40, 52, 53). One study (52) showed that Biodentine had greater antibacterial activity than TheraCal LC, with no significant difference (*p* > 0.05). Whereas TheraCal LC had significantly (*p* < 0.05) better antibacterial activity in one study (40) and non-significantly (*p* > 0.05) in one study (53). Furthermore, the study by Akin et al. 2024 (35) reported no antibacterial activity for TheraCal LC and Biodentine after the complete setting reaction. Additionally, the antibacterial activity of Biodentine was compared to Dycal in 4 studies (32, 35, 40, 54). Biodentine had significantly (*p* < 0.05) greater antibacterial activity than Dycal at different time intervals in one study (32). In addition, Biodentine was non-significantly (*p* > 0.05) superior in inhibiting bacterial growth immediately and 24 h and significantly (*p* < 0.05) at 1 week in one study (54), whereas, Dycal had significantly (*p* < 0.05) higher antibacterial activity in one study (40) at 24 h and 48 h. The study by Akin et al. (35) revealed no antibacterial activity for Dycal and Biodentine after a complete setting reaction. Furthermore, the antibacterial efficacy was significantly (*p* < 0.05) superior in Biodentine compared to GIC (42), RMGIC (43), DiaRoot BioAggregate (45), NeoPutty (31), and iRoot FS (50). Biodentine had greater antibacterial activity with non-significant differences (*p* > 0.05) compared to MTA Repair HP (44), MTA Biorep (39), Well-Root PT (39), Activa (40), NeoMTA 2 (31), and Calcimol LC (40). Finally, the antibacterial activity was significantly (*p* < 0.05) inferior in Biodentine compared to Theracal PT (53), Rootdent MTA (32), and Zinc polycarboxylate cement (37).

## 4 Discussion

Endodontic failure is generally attributed to the lack of proper cleaning of the root canal system and leakage of bacteria into the periradicular tissues. When the infection persists after endodontic treatment/re-treatment, an apicoectomy is indicated and a root-end filling material is placed to prevent re-infection of the root canal. Therefore, the antibacterial activity of endodontic materials is essential for the treatment success in order to prevent or delay infection and extend the lifetime of restorations. Biodentine, a calcium silicate-based material, has gained popularity in recent years due to its various clinical applications, including root-end filling procedures (69, 70). However, there is a shortage of studies and conflicting results regarding the antibacterial activity of Biodentine (71).

*E. faecalis* is an anaerobic bacterium associated with endodontic failure and persistent periapical infection (72). Although it is not part of the root canal system's microbial flora (73), it is discovered in the oral cavity from contaminated food. It was suggested that this bacterium penetrates the root canal system through several ways, including the lack of coronal seal after root canal treatment, dentinal fractures, carious progression in contiguity with the root canal system during pulp necrosis or inflammation, bloodstream, and through root fracture or lateral canals (74). This species has been reported to exhibit varying degrees of resistance to several antimicrobial agents (75) and intracanal dressings such as calcium hydroxide (76), making its eradication from the root canals challenging. This review focused on the studies that investigated the antibacterial activity of Biodentine against *E. faecalis*, as it is one of the main bacteria and most commonly involved in studies on persistent periapical infections (13). Although a recently published review article (71) identified the antimicrobial efficacy of Biodentine, it provided brief coverage and limited details regarding its specific efficacy against *E. faecalis*.

The current review revealed that Biodentine has good antibacterial activity against *E. faecalis,* with thirty out of 37 included studies reporting positive effectiveness. One study exhibited limited effectiveness, four showed conflicting results, and only two demonstrated negative effectiveness. The outcomes of the included studies appear to have minor discrepancies likely due to their different methodologies such as assessment methods, concentration of the microorganism, and the amount of test materials. The result of ADT is semi-quantitative and depends on the solubility and diffusability of the material within the agar medium (77). Solid endodontic materials may not be diffusible (50). The carry-over effect may also be affected by the insolubility of the material (50). Whereas DCT is a quantitative and reproducible method allowing testing of water-insoluble materials and accurately mimics the contact between the materials and microorganisms (54, 77). It demonstrates the material's bactericidal or bacteriostatic effects regardless of its diffusibility in the medium (78). Therefore, ADT is less sensitive in evaluating the antibacterial activity of calcium silicate-based cement compared to direct culture test (31) and DCT (50), which explains the conflicting outcomes between the ADT and other evaluating methods in the same study.

The antibacterial effect of Biodentine is mainly attributed to its alkalinity and calcium release. The cement hydration process generates a colloidal gel and releases calcium hydroxide, which inhibits bacterial growth. Additionally, as Biodentine sets, its pH rises to 12.5 which prevents bacterial growth and disinfects the surrounding area (46, 79). Moreover, it was reported that Biodentine inhibits microbial adherence, resulting in a strong antibacterial activity (79).

For the persistence of the antibacterial activity of Biodentine at different time intervals, conflicting evidence was reported among the included studies, where 3 out of 13 studies demonstrated statistical similarities over time, 2 demonstrated an increase in antibacterial activity, and 3 demonstrated nearly the same level. One demonstrated an increase followed by a decreased antibacterial activity. While a reduction was reported in 2 studies, and no bacterial inhibition in 2 studies. The antibacterial activity of Biodentine against *E. faecalis* appears to be influenced by both aging time and the medium used, with conflicting results between studies. One study exhibited no difference in antibacterial activity across different aging periods in either water or FBS, while another study found that bacterial inhibition in water leachates increased over time when the medium was not refreshed but decreased when refreshed. Additionally, FBS leachates showed lower antibacterial activity compared to water.

This review revealed that Biodentine was superior to or, at least, as efficacious as several commercial endodontic materials against *E. faecalis* such as MTA [in 8 (31, 41, 42, 48–51, 63) out of 11 studies], MTA Angelus [in 11 (31, 33, 34, 36, 43, 45, 46, 52, 54, 64, 65) out of 13 studies], GIC (42), RMGIC (43), DiaRoot BioAggregate (45), NeoPutty (31), iRoot FS (50), MTA Repair HP (44), MTA Biorep (39), Well-Root PT (39), Activa (40), NeoMTA 2 (31), Calcimol LC (40), TotalFill (67), and IRM (55, 56, 67). Whereas, the antibacterial activity of Biodentine was inferior to Theracal PT (53), Rootdent MTA (32), and Zinc polycarboxlate cement (37). Furthermore, it was not feasible to reach a summary on the comparative antibacterial effects of Biodentine in comparison to MTA Plus, CEM, ERRM, TheraCal LC, and Dycal because of the conflicting results among the included studies.

### 4.1 Limitations and strengths of the study

The current evidence indicates that Biodentine is a superior material in endodontic treatment with potent antibacterial activity against *E. faecalis*. However, this evidence lacks a sufficient clinical base to support Biodentine for routine use in inhibiting *E. faecalis* growth. All the included studies and collected data were *in vitro*, which is unreliable in determining Biodentine's clinical potential. Although *in vitro* studies with high-quality and well-designed methodology offer helpful solutions for clinical issues, randomized controlled clinical trials reveal the most dependable and robust outcomes (80). Another limitation of this review was the use of various antibacterial assessment methods (such as ADT, DCT, antibiofilm assay, tube dilution method, bacterial adhesion assay, broth dilution method, carry-over effect test, direct culture test, and dentine block model), and heterogeneity in their procedures among the studies [such as bacterial strains, evaluation times, amount of test materials sample, and various measurements (CFUs and OD) used in DCT and antibiofilm assay]. The lack of standardization and evaluation criteria among the included studies caused the cross-study comparison to be hard to execute, and ultimately conducting a meta-analysis was not practical (81). It is important to highlight the necessity of developing standardized methods for the evaluation of antibacterial activity. A further limitation was that some studies examined the antibacterial activity of Biodentine on planktonic bacteria (such as tube dilution method, broth dilution method, direct culture test, and carry-over effect test). This model does not closely resemble *in vivo* or clinical circumstances because the bacteria are present as complex biofilm communities in the oral cavity (82). Contrastingly to planktonic cells, biofilm structures provide resistance against antimicrobial agents as bacteria are embedded in a hydrated polymeric matrix that serves as a shield to protect bacterial growth (83, 84). The methodological quality evaluation in this review indicated that the findings were supported by studies with medium to high risk of bias (low quality). Lastly, even though the protocol of this study was not registered, the PRISMA guidelines were strictly followed.

This review provides several key strengths that enhance the validity and reliability of the study, including a comprehensive literature search in reputable databases that ensures a broad coverage of relevant studies. The review also employed clear eligibility criteria and rigorously evaluated the risk of bias using the Modified CONSORT checklist items for pre-clinical *in vitro* studies on dental materials, to ensure the reliability of the findings. In addition, it offers a comparative analysis of Biodentine's antibacterial effect relative to other commercial endodontic materials against *E. faecalis,* providing a more relevant understanding of Biodentne's relative efficacy in clinical practice. Finally, the main finding of this study, the strong antibacterial activity of Biodentine in inhibiting the growth of *E. faecalis*, was strongly supported by a substantial number of studies demonstrating favorable effects against *E. faecalis*. This consistent evidence highlights the Biodentine's significant potential as an endodontic material and supports the acceptance of the null hypothesis of the review.

### 4.2 Implications of the results for biodentine's clinical practice

The results of this systematic review suggest that Biodentine's strong antibacterial activity against *E. faecalis* has significant implications for clinical practice. Clinicians can confidently use Biodentine to help prevent bacterial infection, promote healing, and improve treatment outcomes, particularly in cases with persistent or resistant infections. In addition, it may help prevent re-infection and treatment failure in these cases. Its use could also aid in reducing inflammation, leading to faster recovery time and fewer post-treatment complications, such as post-operative infections or flare-ups, thereby enhancing endodontic treatments. Its comparability or superiority to other materials, such as MTA and GIC, supports its potential as a preferred choice in clinical applications, including root-canal sealing and apical surgery. This is further reinforced by its favorable biological and physicochemical properties, as well as its cost-effectiveness. The findings could also guide future clinical trials, inform material selection, and help clinicians optimize infection control in endodontic procedures for better patient care.

Overall, the current review provides clear evidence that Biodentine has a strong efficacy in inhibiting the growth of *E. faecalis*. In addition, most of the studies supported that the antibacterial activity of Biodentine increases or stays nearly at the same level over time. Furthermore, Biodentine was superior to or, at least, as efficacious as MTA, MTA Angelus, GIC, RMGIC, DiaRoot BioAggregate, NeoPutty, iRoot FS, MTA Repair HP, MTA Biorep, Well-Root PT, Activa, NeoMTA 2, Calcimol LC, TotalFill, and IRM.

## 5 Conclusion

Considering the limitations of this systematic review, there is accumulating evidence of the antibacterial activity of Biodentine against *E. faecalis* in the context of endodontics. However, randomized clinical trials with well-designed and robust methodologies are required to provide information about its clinical behaviour. The findings were supported by studies with medium to high risk of bias (low quality). There is a demand for low-risk-of-bias studies to further evaluate the finding's reliability. Furthermore, it is highly suggested to develop standardized methods to evaluate the antibacterial efficacy of endodontic materials in *in vitro* investigations.

## Data Availability

The original contributions presented in the study are included in the article/Supplementary Material, further inquiries can be directed to the corresponding authors.
